# Grouting technology and construction schemes of a tunnel in aeolian stratum: a case study of Shenmu No. 1 tunnel

**DOI:** 10.1038/s41598-021-03021-4

**Published:** 2021-12-07

**Authors:** Fei Ye, Xing Liang, Xiaoming Liang, Wenjun Zhang, Chang Liu, Haolan Feng

**Affiliations:** 1 School of Highway, Chang’an University, Xi’an, Shaanxi China; 2 School of Civil Engineering, Tianjin University, Tianjin, China

**Keywords:** Civil engineering, Engineering

## Abstract

The naturally formed aeolian sand dunes in northern Shaanxi exhibit unique engineering characteristics. Several challenges, such as the poor self-stabilization ability of the surrounding rock, difficultly in injecting grout, and insufficient construction experience, restrict the construction of road tunnels under this stratum. Therefore, in this study, a case study of the Shenmu No. 1 tunnel was conducted to investigate the engineering characteristics of aeolian sand tunnels, compare the grouting effects of commonly used grouting materials, and discuss the reinforcement effects of different construction schemes in aeolian sand tunnels. Based on a field grouting test, it was determined that it is difficult to inject ordinary cement grout into an aeolian sand layer. Furthermore, it was determined that superfine cement grout and modified sodium silicate grout can be injected, but the former exhibits a poor reinforcement effect. Additionally, results of numerical analysis indicated that an approach based on a concept of “horizontal jet grouting pile + benching partial excavation method with a temporary invert” is suitable for the construction of tunnels in aeolian sand in China.

## Introduction

Deserts and sandy land cover a total area of 1.54 million square kilometers in China. This accounts for approximately 16% of the total territorial area of China. They are distributed in Xinjiang, Inner Mongolia, Qinghai, Gansu, and other northwestern regions, accounting for 95.37% of these areas. An aeolian sand series results from the flow and movement of sand, and is characterized by its strong fluidity and ease of slip^1–4^. Aeolian sand is the main landform in the desert areas. Owing to its engineering nature, transportation construction in desert areas has always been extremely difficult. Since the 1990s, China has completed aeolian sand tunnel projects such as the Xingshumao Tunnel, Jingpeng Tunnel, and Qiansongba Tunnel. However, given that aeolian sand exhibits strong fluidity and significantly different engineering properties from those of general strata, the factors that should be considered during the design phase, construction phase, and accident management are relatively complicated. Accordingly, progress on projects in aeolian sand strata has been slow.

Many studies have been conducted on aeolian sand^5–8^. However, given that projects related to aeolian sand are relatively rare, the contributions of the current research are focused on the physical and mechanical properties of aeolian sand. Zhang examined the physical and chemical properties of aeolian sand in the Mu Us Desert and its engineering applicability^9^. Liu systematically analyzed the feasibility of applying aeolian sand to highways from the perspectives of strength, compatibility, and stability^10^. Wang identified the geochemical characteristics of surface fine-grained sediments in the mid-latitude deserts of Asia^11^. Gao conducted a series of indoor mechanics experiments to analyze the effects of moisture content and density on the mechanical properties of aeolian sand, and summarized the mechanical characteristics and parameter change laws of aeolian sand^12^. Zhang conducted experimental and numerical analyses to study the effect of sand deposition on a track structure^13^. Li studied the utilization of aeolian sand for concrete production and analyzed its workability and mechanical properties^14^.

Factors, such as sand leakage and sliding in the aeolian sand stratum, can easily lead to safety accidents such as large deformations of supports, collapses, and roof falls. Hence, the construction of aeolian sand tunnels is considered extremely difficult and risky, and it can essentially act as a bottleneck in engineering construction^13,15^. Additionally, there is no special and complete construction plan for aeolian sand. The responses to corresponding emergencies are insufficient. More generally, to date, a well-formed, systematic, and available construction mode does not exist. Currently, the number of aeolian sand tunnels that are being built (or have been built) is relatively small. In most cases, the excavation can be barely completed, and it usually requires a certain means of strong support. However, given the increase in the number of tunnels that are being constructed in aeolian sandy strata in China, the shortcomings (such as insufficient construction experience and the lack of a well-formed systematic construction mode) have become increasingly prominent. Therefore, relevant research is urgently required to guide the design and construction of tunnels in aeolian sand strata.

Many studies have been conducted on this worldwide^16–19^. Jin et al. adopted a pipe shed advanced support and observed that as the effective range of the one-time support increases, the integrity of the support increases and safety of the construction process increases^20^. Qiu et al. analyzed the mechanical behaviors of aeolian sand tunnels during the construction process using fast Lagrangian analysis of continua 3D (FLAC^3D^) based on field monitoring data^21^. The research primarily focused on an in-depth analysis of the supporting effects of horizontal jet grouting piles and the effects of construction steps on surrounding rock deformations under the action of support. Yan et al. identified and assessed the risk sources of collapse in aeolian sand tunnels based on a fuzzy evaluation method, expert investigation method, and analytic hierarchy process, and determined risk indicators^22^. They indicated that construction/design factors and the choice of advanced support were the most important one-level and two-level factors, respectively. Wang et al. used the discrete element software UDEC to analyze the reinforcement effects of vertical jet grouting piles and horizontal jet grouting piles in aeolian sand tunnels^23^. Many researchers have examined the feasibility of jet grouting in tunneling projects^24^. Shen et al. proposed a framework for incorporating a bidirectional long short-term memory and data sequencing to predict the diameters of jet grouted columns in soft soil in real time^25^.

However, relatively few studies considered the applicability of grout and advanced grouting technology in aeolian sand, especially based on in-situ tests. Moreover, the construction technologies have not been compared in detail and an optimum technology has not been identified. Accordingly, in this study, Shenmu No. 1 tunnel is used as an example to conduct research on a construction technology for shallow highway tunnels in aeolian sand strata. This study aims to solve technical problems in aeolian sand tunnel grouting and construction technology, and to ensure the construction safety and engineering quality of aeolian sand tunnels. Hence, these issues are of great engineering practical significance.

## Background

### Geological characteristics of aeolian sand for Shenmu No. 1 tunnel

#### Physical properties

The Shenmu No. 1 tunnel in the Yu-Shen highway is located on the edge of the Mu Us Desert. The starting and ending pile numbers of the left line are ZK90 + 998–ZK91 + 360, and the length is 362 m. The starting and ending pile numbers of the right line are K90 + 993–K91 + 345, and the length is 352 m. The aeolian sand in this location is generally brownish-yellow, loose, and slightly wet. It is primarily composed of feldspar (73%) and fine quartz sand (23%), followed by silt sand and silty soil, which are concentrated at the tunnel exit and in the surface above the tunnel. They exhibit a covering layer thickness in the range of 15–35 m. The main physical and mechanical indexes, as obtained through field experiments, are listed in Table 1, and the particle size distributions are listed in Table 2. In this study, the method adopted for separating particles corresponded to a sieving method (Test Methods of Soils for Highway Engineering JTG E40—2007). The particles were mostly composed of fine sand (0.075–0.25 mm), followed by very fine sand (0.01–0.075 mm), and medium sand (0.25–0.5 mm). Specifically, sand with a size greater than 0.5 mm was rare. The uneven coefficient $C_{u}$ was 3.5, and the curvature coefficient $C_{c}$ was 0.64, thereby indicating poor gradation.

**Table 1:** Physical and mechanical indexes of aeolian sand.

Sampling location	Water content (%)	Natural void ratio	Natural bulk density (kN/m^3^)	Cohesion (MPa)	Internal friction angle	Modulus of deformation (MPa)	Permeability coefficient (cm/s)
ZK91 + 232	4.4	0.447	17.155	0.011	26.88	21.0	3.56
K91 + 240	4.8	0.456	17.165	0.012	27.65	22.0	3.50

Tests for physical and mechanical indexes were conducted based on Test Methods of Soils for Highway Engineering (JTG E40—2007)^26^.

**Table 2:** Gradation of grain of aeolian sand (%).

Sampling location	Grain size (d/mm)				
	> 1.0	1.0–0.5	0.5–0.25	0.25–0.075	< 0.075
ZK91 + 232	0	2.1	19.7	47.6	30.6
K91 + 240	0	4.3	18.5	47.9	29.3

Based on the physical and mechanical indexes and gradation of the aeolian sand, it can be observed that its particles exhibit the characteristics of a small cohesive force, poor gradation, low compressibility, inclination to disturbances owing to excavation, strong water permeability, and a relatively low shear strength.

#### Mechanical properties

##### Compressibility

Aeolian sand exhibits a single-grain structure, and its compression is primarily dependent on the rearrangement and fragmentation of the particles. Under the action of low pressure, the particles slip and roll, making the soil denser and more stable. The amount of compression is determined by the frictional resistance between the particles against displacement. The better the gradation and higher the density, the greater the resistance and the smaller the compression deformation. The compression process of aeolian sand begins with almost instantaneous sinking, followed by long-term deformation at a deceleration rate. This denotes the process of gradually adjusting the position of the particles to overcome resistance. The compressibility coefficient of aeolian sand is generally small (less than 0.1 MPa^−1^), showing low compressibility.

##### Strength characteristics

The strength characteristics, especially the density values under various water contents, play an important role in grout injection. Under the same water content, when the dry density of aeolian sand increases, the corresponding internal friction angle and cohesion increase. Under the condition of constant dry density, when the water content decreases, the internal friction angle and cohesion decrease. Furthermore, as the dry density increases, the shear strength increases. Under constant values of dry density and vertical pressure, the water content slightly affects the shear strength. Specifically, the difference in the shear strength value is approximately 10 kPa. Under the influence of capillary force with a low vertical pressure, when the water content is less than a certain value (14%), the shear strength increases as water content increases. Furthermore, when the water content is higher than a certain value (14%), the shear strength decreases with an increase in water content.

### General information of Shenmu No. 1 tunnel

**Figure 1:**
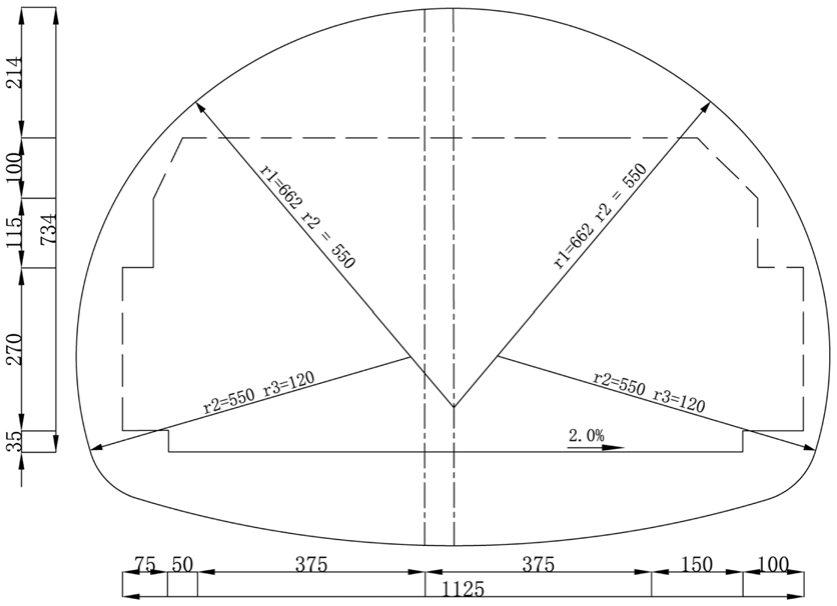
Horizontal profile of Shenmu No. 1 tunnel (unit: cm).

The Shenmu No. 1 tunnel adopts the form of two independent single-hole tunnels separated from the upper and lower sides. The tunnel was constructed using a shallow tunneling method, with a steel arch, steel mesh, and shotcrete as the initial support. Furthermore, molded concrete was used as the secondary lining. The horizontal profile of the tunnel is shown in Fig. 1.

Based on the results of a ground survey, drilling, and geophysical prospecting, Quaternary Holocene aeolian sand (Q_4_^eol^) was observed as overlying the tunnel site area and Triassic fine sandstone was observed as underlying the tunnel. The soil layers from top to bottom are as follows: aeolian sand layer followed by fully weathered–strongly weathered fine sandstone.

## Grouting technology in aeolian sand stratum

**Figure 2:**
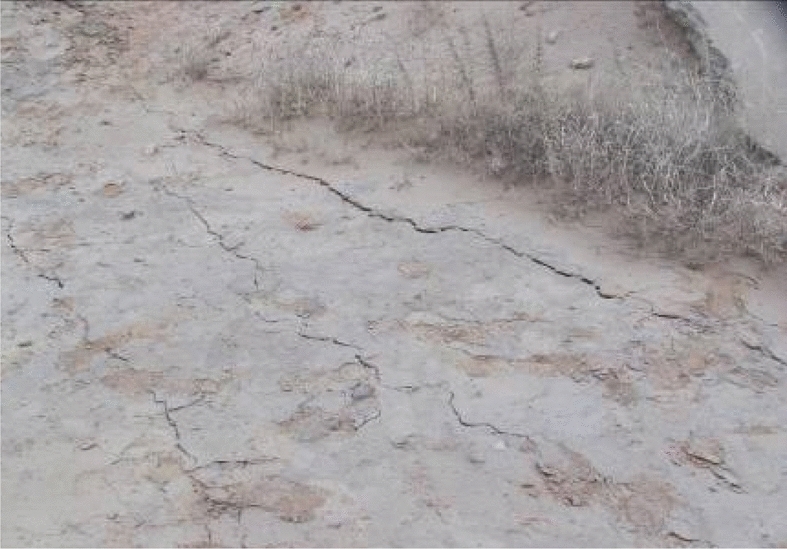
Cracks on the surface.

**Figure 3:**
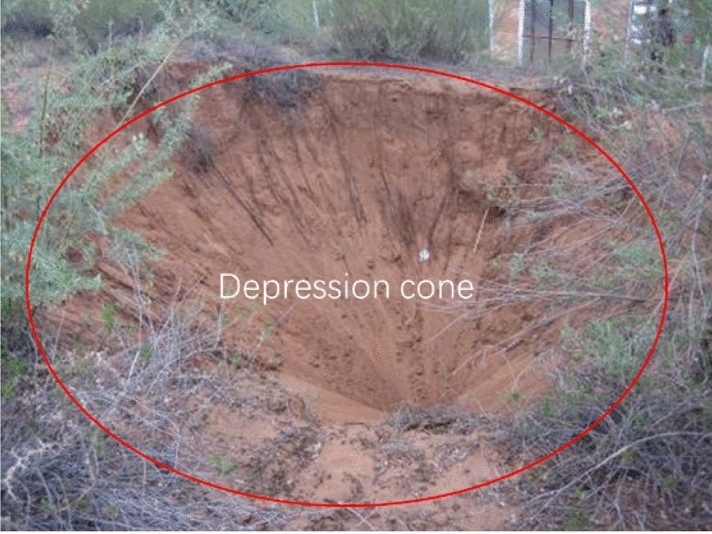
Depression cone on the surface.

An advanced small pipe grouting support method was adopted in the early construction stage of the Shenmu No. 1 tunnel. This was combined with a four-step excavation method. During the construction, severe sand leakage and sand sliding occurred in the tunnel face and side walls. The primary support sank overall and invaded the tunnel clearance, and a depression cone (up to 10 m in diameter) and cracks (up to 3 cm wide) were observed on the surface (Figs. 2, 3). Specifically, two potential causes were considered for the sand leakage and development of cracks: (1) the sliding surface of the sand body extended to the ground, indicating that the sliding surface of the sand body can potentially exceed the scope of the advance support and pre-reinforcement; and (2) the grouting of the advance support failed to achieve the designed reinforcement effect, and the soil above the vault slid into the tunnel along the gap between the grouting pipes.

In summary, the key problem during the construction of aeolian sand tunnels is sand leakage. This section discusses field grouting tests conducted using different grouting methods and materials. The geological characteristics, grouting mechanism(s) of aeolian sand strata, and existing problems in the grouting process are summarized and examined to promote further development of grouting technology, expand its application range, and solve technical problems in similar projects.

### Field grouting test

**Figure 4:**
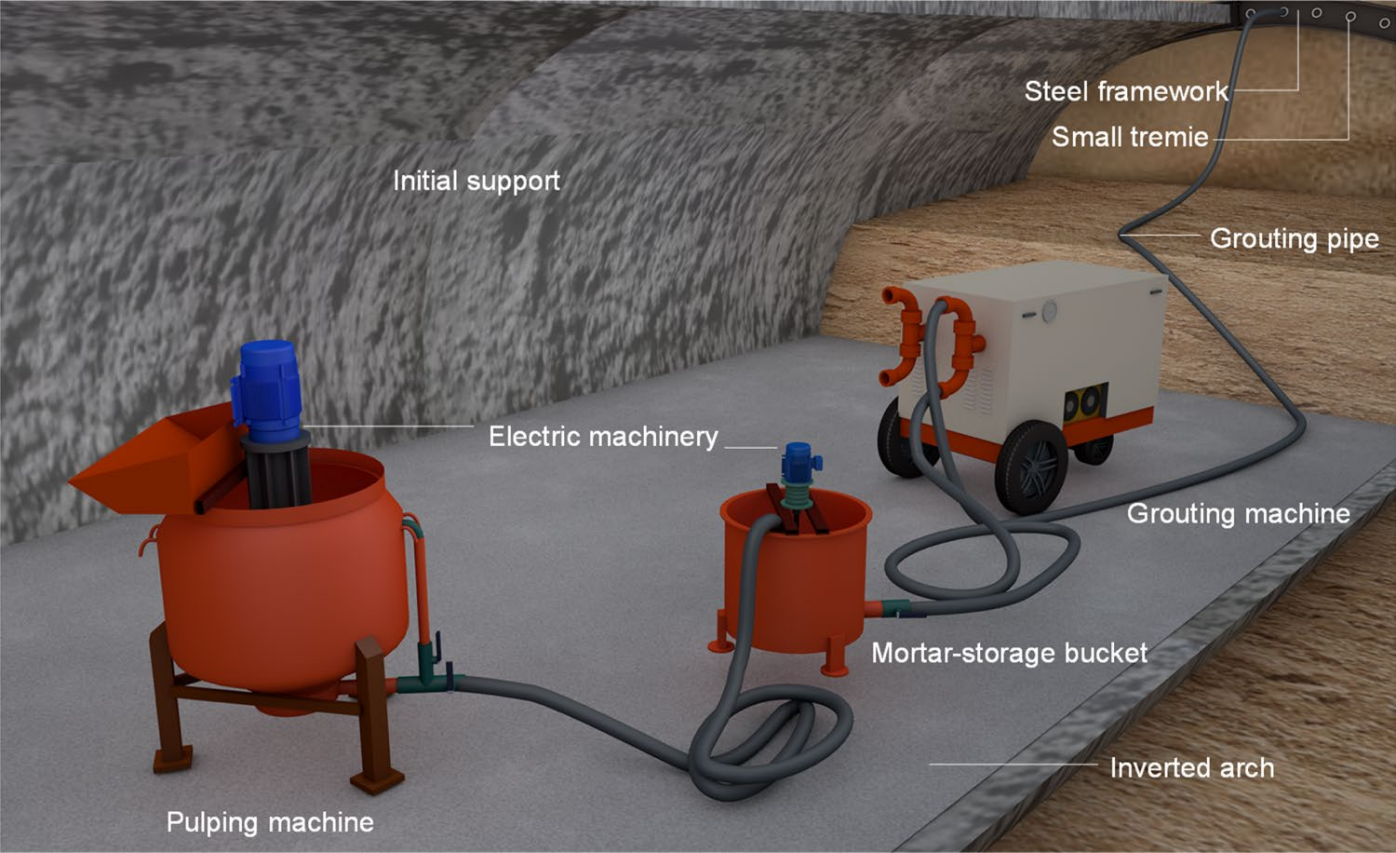
Sketch map of grouting inside the tunnel.

With respect to the geological conditions of the Shenmu No. 1 tunnel, the effect of grouting is a key factor in controlling the surface settlement and ensuring construction safety. The simple application of an engineering analogy method and/or a semi-empirical engineering method to determine the grouting parameters can lead to great risks. Therefore, to enhance the understanding of the grouting characteristics of the formation, obtain the necessary technical and economic data, and demonstrate the rationality of the grouting scheme, a representative section should be selected for field grouting tests before grouting construction (Fig. 4).

Based on the understanding of the basic properties of various grouting materials, in this study, the test combined the engineering geological conditions, hydrogeological conditions, and criticality class of the grouting in the aeolian sand section of the Shenmu No. 1 tunnel. A penetration grouting method was selected to evaluate the advantages and disadvantages of each grouting material and grouting method via the analysis of test data and to optimize the grouting ratio scheme.

#### Ordinary Portland cement grout

**Figure 5:**
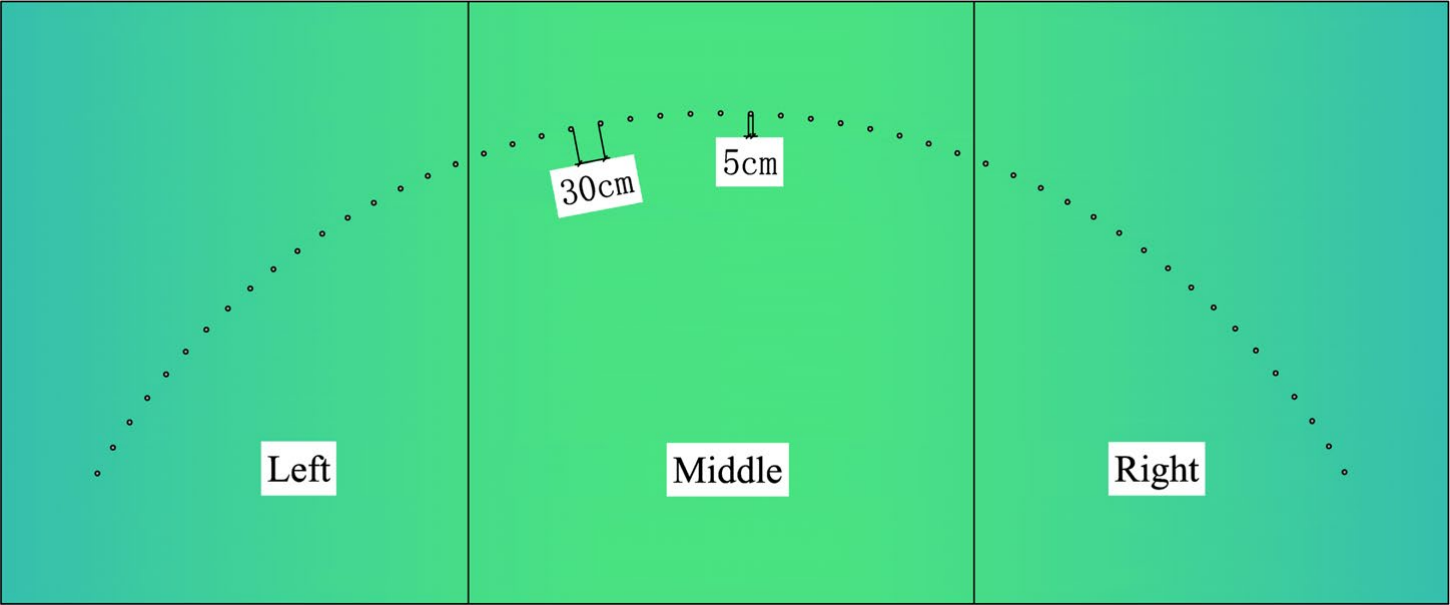
Layout of grouting pipes in vault area.

At K91 + 225 of the right tunnel, a series of φ50 small pipes were installed outside the excavation contour line of the upper part of the tunnel. The circumferential spacing of the pipes was 30 cm. A total of 51 pipes were installed in three sections with a range of 120° in the upper tunnel profile, i.e., 17 pipes were located in each pilot area. Specifically, the entire section was divided into three pilot areas on the left, middle, and right (Fig. 5). Three grouts with different water–cement ratios, i.e., $m_{w} :m_{c}$ = 0.8:1, 1:1, and 1.5:1, were adopted to conduct in-situ tests in the three pilot areas. The grouting construction was completed on the first day, and the excavation was conducted on the next day to examine the grouting effect.

During the test, the value of the grouting pressure gauge increased rapidly and reached the final pressure value within a short time (10–20 s). Once the grouting volume remained constant, it was observed that the final volume was significantly smaller than the design grouting amount. The grout failed to spread and did not satisfy the design reinforcement requirements. After excavation, it was revealed that there was no penetration of grout into the aeolian sand layer. However, grout clusters with diameters in the range of 2–10 cm were observed around the holes of the small tremies. These grout clusters failed to form a whole reinforced body. Therefore, the sand leakage continued to remain a serious problem during the excavation. Although the grouting amounts of the three grouts were different, the reinforcement effects were not significantly different. Ordinary Portland cement grout does not easily permeate in to aeolian sand formations because ordinary Portland cement exhibits a large particle size. Hence, it is difficult to inject ordinary Portland cement into an aeolian sand layer with pores of 10 μm. Furthermore, the cement particles are suspended in the slurry. Thus, even if the particles are smaller than the pores of the sand layer, owing to the filtering effect of the sand layer, the penetration range is extremely small. Furthermore, sometimes the slurry consolidates around the grouting holes. Therefore, based on the principle of particle size matching, this grout was considered to be only suitable for broken rock layers or coarse gravel sand layers, and it was not considered to be suitable for aeolian sand layers.

#### Superfine cement grout

**Figure 6:**
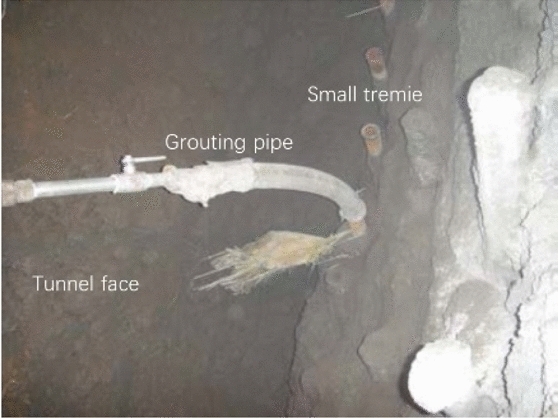
In-situ test of superfine cement grout.

At K91 + 245 of the right tunnel, ordinary cement was modified to superfine cement for testing. Three grouts with different water–cement ratios, i.e., $m_{w} :m_{c}$ = 1:1, 1.5:1, and 2:1, were adopted to conduct in-situ tests in the three pilot areas (Fig. 6).

During the test, the value of the grouting pressure gauge increased rapidly and reached the final pressure value in a relatively short time (20–30 s). Once the grouting volume was constant, it was determined that the final volume was lower than the design grouting amount. The grout failed to spread well, and it did not satisfy the design reinforcement requirements. After excavation, heterogeneous penetration of grout was observed in the aeolian sand layer and the radius of penetration was small. Similar to the results of the test involving the ordinary Portland cement grout, grout clusters with diameters of 2–10 cm were observed around the holes of the small pipes. Although the amount of grouting with respect to the three grouts varied, the reinforcement effects were not significantly different. The superfine cement grout exhibited a small particle size, with an average particle size of 4 μm. Theoretically, it can be injected into the aeolian sand layer with a pore size of 10 μm. However, the difference in the reinforcement effect, when compared with that of ordinary cement grout, was not high because the superfine cement grout was prone to sediment and self-stability was poor. Superfine cement grout can penetrate aeolian sand formations and can be potentially adopted as a high-quality grouting material. However, its stability and grouting technology should be further improved to utilize its advantages optimally.

#### Modified sodium silicate grout

**Figure 7:**
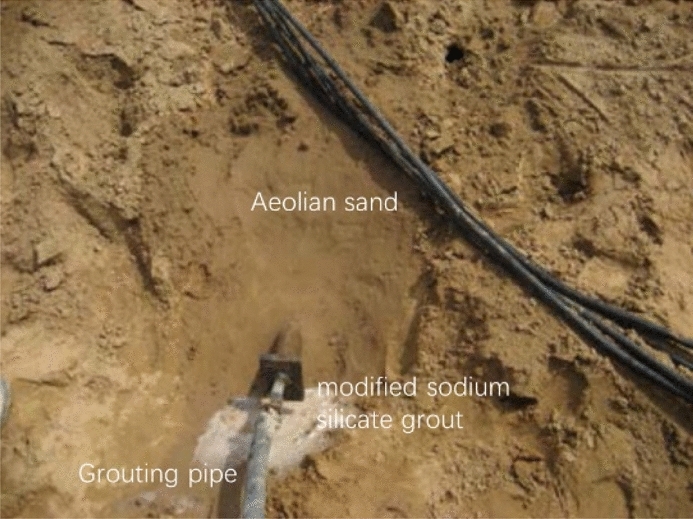
In-situ test of modified sodium silicate grout.

After the superfine cement grouting test was completed, a modified sodium silicate grouting test was selected for the roadbed outside the exit of the right tunnel to avoid affecting the normal construction in the tunnel. Specifically, φ50 small pipes were placed horizontally along the side slope of the roadbed. The pipe length was 4.5 m and spacing was 30 cm for a total of 15 pipes. The test was divided into three groups with five small pipes in each group (Fig. 7).

During the test, the injectability of the grout was significantly improved, and the permeability was better than those observed in the aforementioned two grouting tests conducted in the aeolian sand formations. However, the gelation time of the modified sodium silicate grout was difficult to control and the grouting process was complicated. Hence, this led to a lower grout volume than the design grouting amount.

After excavation, it was revealed that there was uniform grout penetration in the aeolian sand formation. Grout clusters, with diameters of 8–10 cm, were observed around the holes of the small pipes. The strength of these clusters was extremely low such that they can be broken via pressure subjected by hand. Furthermore, the grout condensed into white flocs after it was exposed to air for a certain period of time.

**Figure 8:**
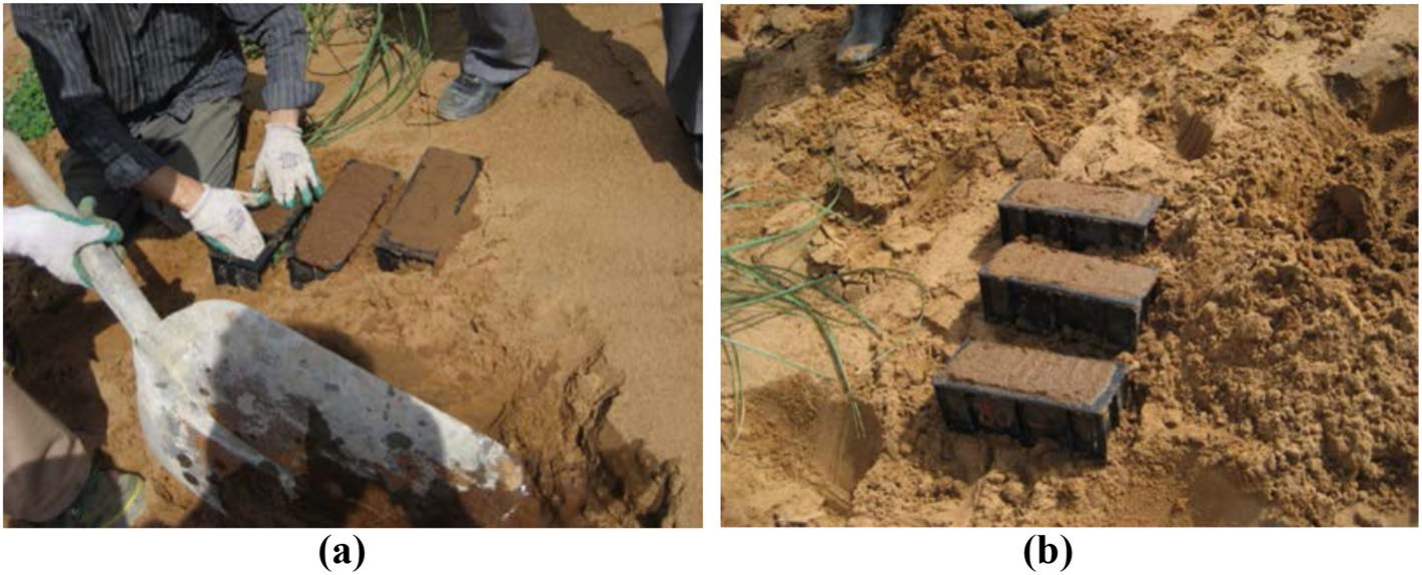
Specimen of reinforced modified sodium silicate grout.

A reinforced modified sodium silicate grout specimen was developed for indoor testing (Fig. 8a,b). The physical and mechanical indices of the reinforced modified sodium silicate grout are listed in Table 3.

**Table 3:** Physical and mechanical indexes of reinforced modified sodium silicate grout.

Water content (%)	Unit weight (N/m^3^ × 10^4^)	Coefficient of compressibility (MPa ^-1^ × 10^–3^)	Modulus of compression (MPa)	Internal friction angle (°)	Cohesive (MPa)	Void ratio
17.33	1.24	1.3	13.15	25.31	0.05	0.751

The sand specimen was cured in air for two days. Its uniaxial compressive strength was 0.2 MPa, and its permeability coefficient was 6.83 × 10^–6^. Thus, it was essentially an impermeable body. Additionally, the durability values of the sand specimen varied after curing in water, air, and buried sand. The sand specimen was buried in water and sand layers, and its strength did not decrease after 3 months. After curing in air for one day, white crystals were observed on the surface of the specimen. Furthermore, the surface was loose and peeled off after one week.

### Analysis of grouting effect

#### Injectability of cement grout under different water–cement ratios

Increasing the fluidity of a grout is an effective measure for improving its permeability. One commonly used method for cement grout involves increasing the water–cement ratio. This is generally based on using high water–cement ratio cement suspension, especially for grouting in smaller cracks, for improving the fluidity and dispersion of the grout. As shown in Table 4, when the water–cement ratio of the superfine cement grout was 1:1, the grouting volume was 16 L, and when the water–cement ratio was 1.5:1, the grouting volume was 28.5 L. With respect to ordinary Portland cement grout, the small increase was ascribed to the poor injectability of the grout, i.e., the ordinary cement had large particles, which could not penetrate the gaps of the aeolian sand formations.

**Table 4:**
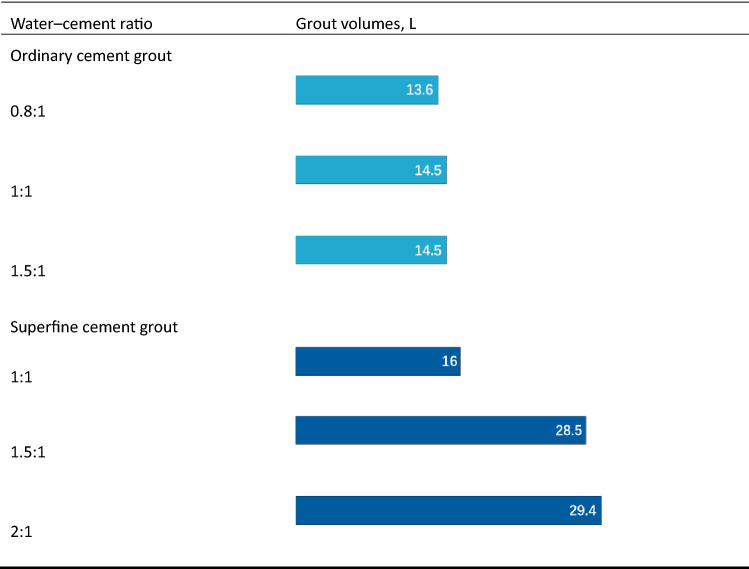
Grout volumes of grouts with varying water–cement ratios.

The deposition of the cement grout with high water–cement ratio occurred throughout the movement in the fissures. As the deposition thickness increased, the pressure transmission and flow velocity changed until a certain section was blocked and closed. The grouting pressure and consistency affected the compactness of the filling body. When the water–cement ratio reached a certain level, the fluidity of the cement grout was no longer significantly improved because the deposition process became a controlling factor and affected the movement of the cement grout in the fissures. To improve the fluidity of the grout, it does not make sense to increase the water–cement ratio to a very high level. As shown in Table 4, when the water–cement ratio of the superfine cement grout increased from 1.5:1 to 2:1, the grouting volume only increases by 4.5%.

#### Analysis of the injectability of different materials in aeolian sand layer

Table 5 shows the grouting volumes of the three different grouting materials for a single pipe. The water–cement ratios of the ordinary cement grout and superfine cement grout were 1:1 and 1.5:1, respectively. With respect to injectability in aeolian sand formations, the chemical grout was stronger than the suspension-type grout, and the grouting volume was three to four times that of ordinary cement grout.

**Table 5:**
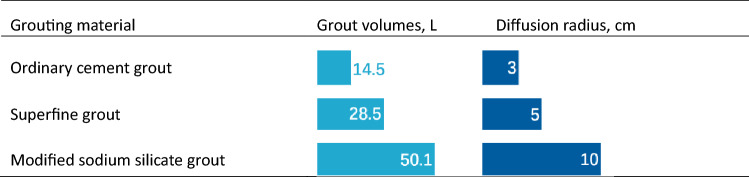
Grout volumes of grouts at varying water–cement ratios.

Table 5 shows the permeability of the modified sodium silicate, which is the highest at up to 10 cm, followed by superfine cement at approximately 4 cm, and ordinary cement grout exhibits the smallest penetration radius of approximately 2 cm, i.e., it is almost non-permeable. This was related to the type and particles of the grout.

Typically, the permeability of a suspended grout composed of solid particulate materials, such as cement, clay, and fly ash, is primarily dependent on the particle size and fluidity of the grout. When the sizes of the cracks or pores are smaller than the diameter of the grout particles, effective grouting cannot be implemented. Hence, it is generally believed that only when the cracks or pores are more than three times larger than the coarsest particles in terms of size can it be used for grouting. If they are smaller than this limit, the coarsest particles can potentially be blocked in the cracks. This in turn leads to a rapid formation of a filter layer. Hence, other smaller particles cannot penetrate. Currently, the ordinary cement produced in China has a particle diameter of approximately 50 μm as the main component, and the thickest particles reach 80 μm. The particle diameter of the aeolian sand of the Shenmu No. 1 tunnel is primarily distributed between 75 and 500 μm, and the distance between particles is 10–20 μm. Hence, particles of ordinary cement grout cannot be injected, and the grout consolidates around the steel pipe and cannot penetrate and diffuse.

The average particle size of the superfine cement grout was 4 μm. Therefore, according to theoretical calculations, it can be injected. However, owing to the small particles of the grout, segregation and sedimentation during the penetration process tended to occur, and grout accumulated at the entrance of the fissure to form a grout layer. Therefore, the penetration depth was limited, and the reinforcing effect was not satisfactory.

When compared with suspended grout, the modified sodium silicate grout exhibited low viscosity and high pourability. However, the gel time significantly affects the permeability. The control of the gel time should be enhanced to obtain an ideal penetration range.

## Construction technology of aeolian sand tunnel

An aeolian sand stratum exhibits low cohesion and poor self-stability and easily collapses during excavation. Hence, construction is difficult. To date, there have been relatively few tunnel projects in the aeolian sand areas of China. Additionally, many issues, such as a lack of construction experience, imperfect construction technologies and methods, and a lack of well-formed, systematic, and applicable construction modes, should be urgently addressed. To discuss the construction technologies for aeolian sand tunnels, we first investigated the construction approaches related to aeolian sand tunnels. The projects that have been completed recently are listed in Table 6.

**Table 6:** Projects in aeolian sand formations in China.

Tunnel	Type of advance support	Construction method	Railway line
New Xingshumao tunnel	Horizontal jet grouting pile, advanced large pipe shed grouting, advanced small pipe grouting	Cross diaphragm (CRD), bench construction	Shenmu-shuozhou Railway
Shahalamao tunnel	Horizontal jet grouting pile	Reserved core soil and bench construction	Baotou-Xi’an Railway
Liuwu tunnel	Advanced large pipe shed grouting	Bench construction	Qinghai–Tibet Railway
New Xiangshawan tunnel	Advanced large pipe shed grouting, advanced small pipe grouting	Reserved core soil and short bench construction	Double track electrified railway tunnels
Huoshatu tunnel	Double-layer advanced small pipe grouting	Short bench construction	New Baotou-shenmu Railway

### Numerical model

According to the majority of the existing numerical studies (e.g., Qiu et al.^21^; Zheng et al.^27^), a finite difference method can effectively solve the problems of construction technology. In this study, FLAC^3D^ was adopted for the analysis. In the establishment and analysis of the numerical model, the following assumptions were considered. First, in the initial stress field of the formation, tectonic stress was not considered and only its geostatic stress was considered. The effects of the advanced support and bolt reinforcement were achieved by increasing the surrounding rock parameters. To fully reflect and compare the effect of the advanced support, the initial support was activated after the excavation calculation was completed.

To ensure sufficient solution accuracy, the general model boundary was three to five times the tunnel diameter, and the semi-infinite boundary was simplified to a finite boundary to eliminate the influence of boundary effects. Simultaneously, the normal displacement of each boundary surface was constrained around the model, the bottom surface was completely constrained, and the top surface of the model was a free surface. Based on this, the width direction (*x* direction) of this model was 100 m, and the height direction (*z* direction) was 36.4 m below the bottom of the invert and 27 m above the vault. Hence, the total vertical direction was 73.8 m. The length direction (*y* direction) was 1 m.

In this numerical simulation, the physical and mechanical parameters of the aeolian sand were determined via experiments. The parameters of the bottom sandstone and each reinforcement ring were determined via a combination of field experiments and literature data (Xiang et al.^1^). The parameters of the shotcrete and molded concrete were determined based on the “Specifications for Design of Highway Tunnels” (JTG D70/2–2014). The detailed parameters are listed in Table 7.

**Table 7:** Parameters for numerical simulation.

Element	Unit weight (kN/m^3^)	Poisson ratio *μ*	Elastic modulus (GPa)	Cohesion (MPa)	Internal friction angle (°)	Depth of reinforcing ring (m)
Aeolian sand	17.2	0.4	0.021	0.011	27	–
Bottom sand	19.1	0.35	1	0.1	27	–
Reinforcing ring by large pipe shed	18.7	0.35	0.24	0.06	30	0.5
Reinforcing ring by small pipe	18.2	0.35	0.13	0.05	30	0.7
Reinforcing ring by horizontal jet grouting pile	19	0.3	4	0.58	35	0.6
Strengthening area by anchors	17.5	0.38	0.04	0.02	28	4.0
Shotcrete	23	0.25	23	–	–	0.3
Secondary lining	25	0.2	31	–	–	0.6

The thickness of the reinforcement ring is based on the radial size of the effective reinforcement shell, which is formed by the advance support and anchor in the formation and by comprehensively considering the design parameters of the large pipe shed, small pipe, horizontal jet grouting pile, and anchor (including the pipe diameter, pipe length, and separation distance) and grouting diffusion radius. By combining the physical and mechanical properties of aeolian sand formation and results of numerical simulation, the soil and secondary lining were adopted in the Mohr–Coulomb model and elastic model, respectively, and solid elements were used for simulation. The initial support adopted the shell element of the structural elements for the simulation.

**Figure 9:**
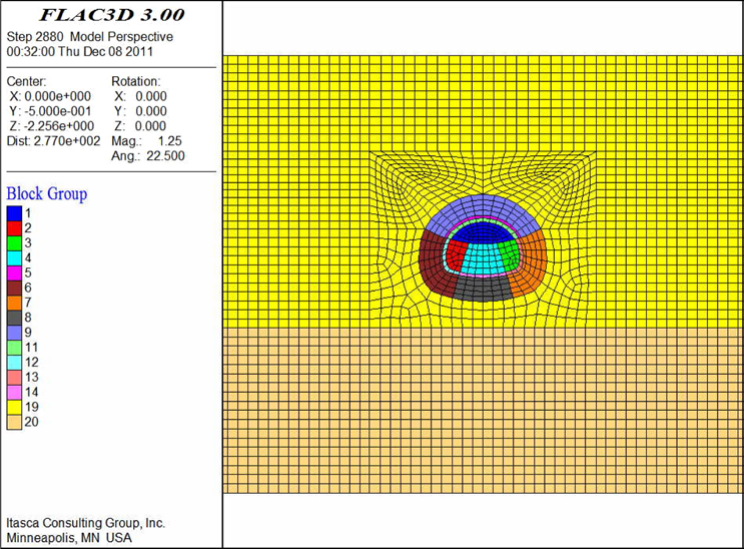
Numerical model of advanced support (FLAC3D 3.0 http://docs.itascacg.com/flac3d700/flac3d/docproject/source/flac3dhome.html).

**Figure 10:**
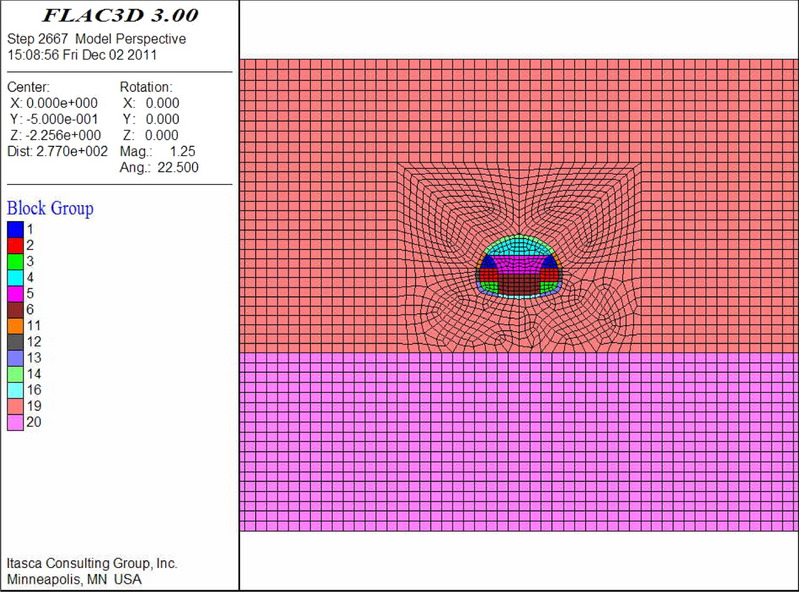
Double-sided pit method (FLAC3D 3.0 http://docs.itascacg.com/flac3d700/flac3d/docproject/source/flac3dhome.html).

**Figure 11:**
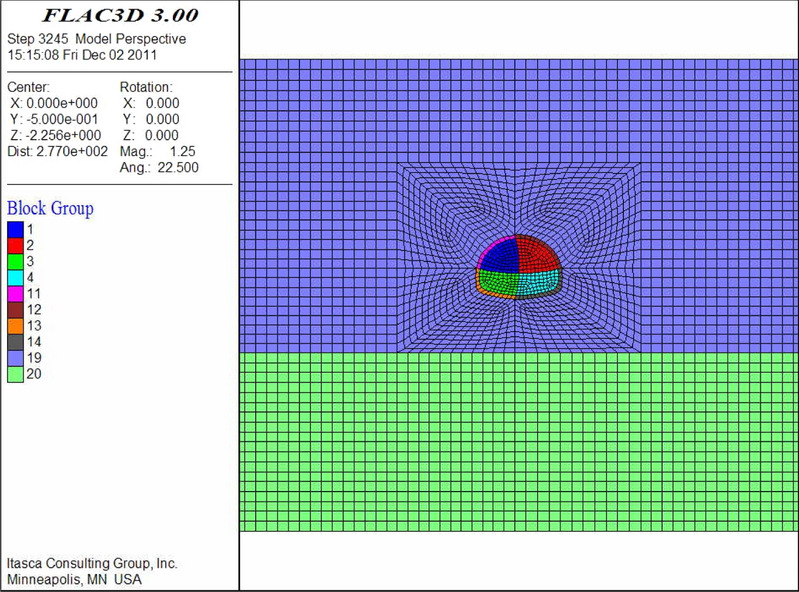
CRD method (FLAC3D 3.0 http://docs.itascacg.com/flac3d700/flac3d/docproject/source/flac3dhome.html).

**Figure 12:**
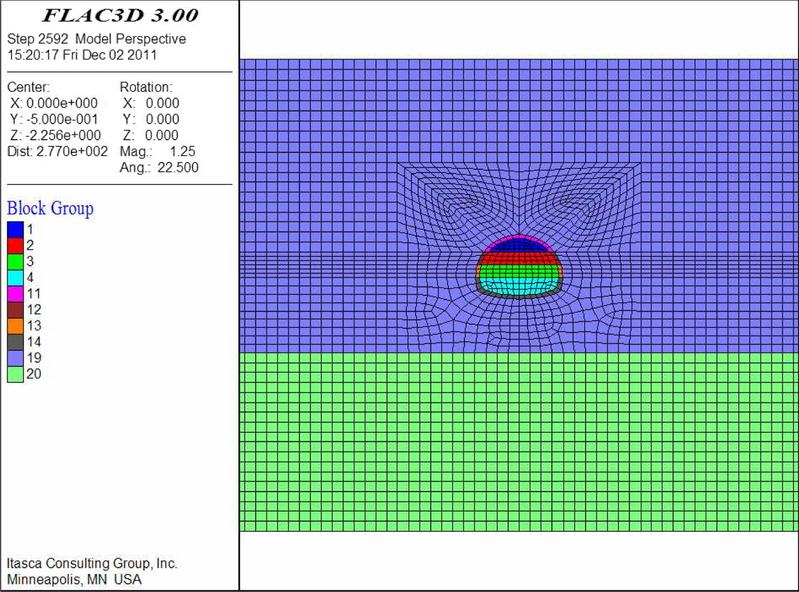
Bench cut method (FLAC3D 3.0 http://docs.itascacg.com/flac3d700/flac3d/docproject/source/flac3dhome.html).

**Figure 13:**
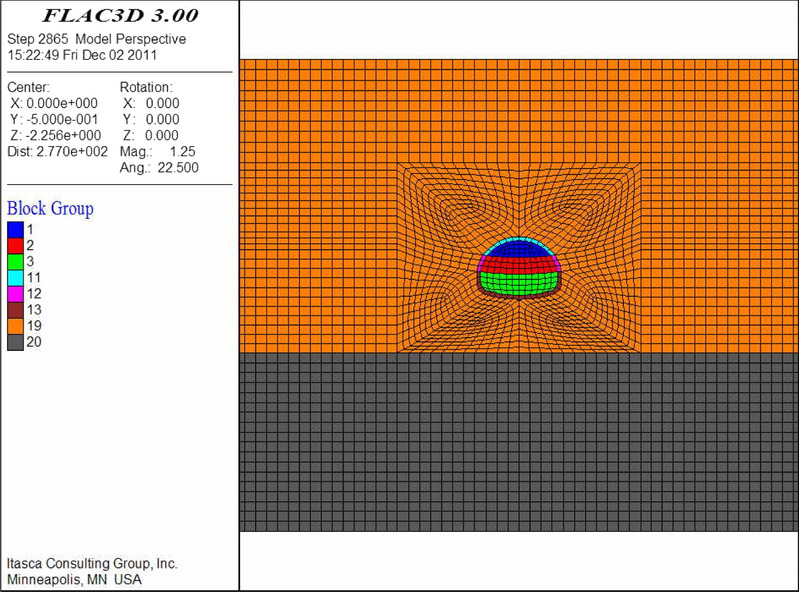
Three-bench method with temporary invert (FLAC3D 3.0 http://docs.itascacg.com/flac3d700/flac3d/docproject/source/flac3dhome.html).

**Figure 14:**
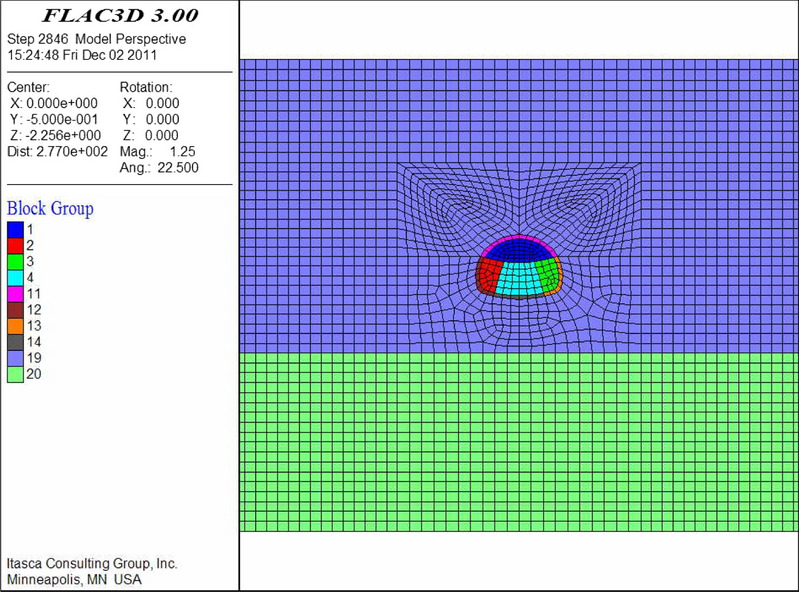
Benching partial excavation method with temporary invert (FLAC3D 3.0 http://docs.itascacg.com/flac3d700/flac3d/docproject/source/flac3dhome.html).

The numerical models are shown in Figs. 9, 10, 11, 12, 13 and 14. Among the models, the difference in the advanced support model is only shown with respect to the size of the reinforcement ring. Therefore, only one of the models is listed as an example.

### Analysis of numerical simulation results

#### Comparison of advanced support methods

Table 8 shows that horizontal jet grouting pile method exhibits the best control effect on surrounding rock deformation and settlement with a vault settlement of 136 mm and surface subsidence of 70 mm. Furthermore, the large pipe shed grouting method and small pipe grouting method exhibit similar control effect on deformation. This is also evident in Table 9 wherein 25% decline in vault settlement of horizontal jet grouting pile method can be achieved when compared with that of non-advance support. Hence, it ranks first among the three advance support methods.

**Table 8:**
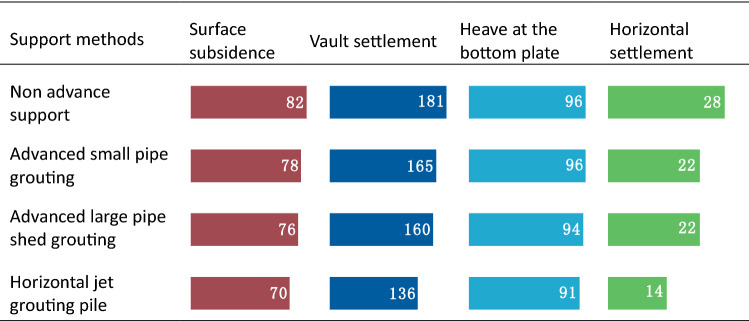
Total settlement of surrounding rock at different support methods (unit: mm).

**Table 9:** Percentage decline in vault settlement at different support methods.

Support methods	Percentage decline in vault settlement at different support methods
Non advance support	–
Advanced small pipe grouting	8% when compared with non-advance support
Advanced large pipe shed grouting	12% when compared with non-advance support, 3% when compared with advanced small pipe grouting
Horizontal jet grouting pile	25% when compared with non-advance support, 18% when compared with advanced small pipe grouting, 15% when compared with advanced large pipe shed grouting

In terms of internal force, Table 10 shows that horizontal jet grouting pile method exhibits the lowest bend moment and highest axial force, while the data of other two advance support methods rank them between the horizontal jet grouting pile method and non-advance support method. Hence, an increase in the advanced support leads to an increase in the axial force of the initial support and a decrease in the bending moment.

**Table 10:**
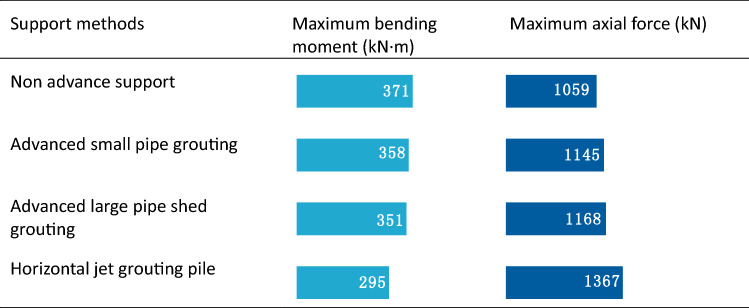
Internal force of primary support at different support methods.

Table 11 shows that the maximum tension stress and maximum compression stress of four methods are approximately equal. An increase in the stiffness of the advanced support leads to an increase in the compressive stress of the second lining.

**Table 11:**
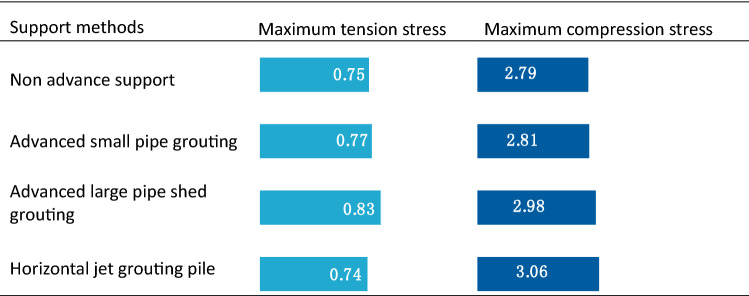
Stress of secondary lining at different support methods (unit: MPa).

In actual projects, a complicated construction technology and long construction period is used for the horizontal jet grouting pile, but its sand-fixing effect, deformation, and settlement control effects are better than those of advanced small and large pipe sheds. Hence, it can effectively solve the technical problems in the construction of aeolian sand tunnels while ensuring the stability of the surrounding rock and construction safety. When compared with horizontal jet grouting piles, the construction technology of advanced large pipe sheds is relatively simple and the construction period is relatively short. Hence, it satisfies the practical requirements for many projects. The key to its successful application in aeolian sand tunnels is the grouting effect. The annular distance and length of the small pipe should be reasonably determined based on the diffusion range and sliding surface of the sand body.

#### Comparison of construction methods

##### Double-sided pit method

The numerical simulation results indicate that the construction process of the double-sided pit method exhibits many divisions during the construction process. This in turn lead to large disturbances. The full-section closure time of the primary support is long, but each section is closed immediately after excavation. Therefore, the deformation due to construction is low, the ability to control the surrounding rock deformation is strong, and the construction safety is high. It should be noted that the structure is subject to complex forces and part of the structure bears a relatively high force. The following points should be noted during the construction.

1. The vault settlement due to the excavation of the upper part of the middle head pit accounts for approximately 65% of the final settlement. Furthermore, attention should be paid during construction and temporary support should be added, when necessary, to control the settlement of the vault in a timely manner.
2. During construction, the primary support bears large forces locally. Thus, the deformation monitoring and observation of the primary support should be strengthened. Meanwhile, reinforcement should be performed, when necessary, to prevent local damage.

##### CRD method

The analysis of the numerical results from CRD method indicate that the CRD method adopts partial construction and adds temporary support. Its ability to control the surrounding rock deformation is relatively strong, the deformation is low, and the construction safety is high. However, the structural force is also relatively high and complex. When compared with the double-sided pit method, the CRD method exhibits a larger construction area, and the deformation due to the construction is also relatively high. Additionally, the vertical temporary support is subject to higher stress during construction. Hence, monitoring and protection should be strengthened.

##### Bench cut method

The numerical results of the bench cut method indicate that the method exhibits poor control ability with respect to the surrounding rock deformation. This in turn leads to wide distribution area of the plastic zone. The following points should be noted during this type of construction.

1. The vault settlement due to the construction of the upper bench accounts for the largest proportion of the final settlement (approximately 75%).
2. During construction, stress concentrations are likely to occur at the bottom corners of every bench. These corners can be reinforced by grouting or by adding lock-foot anchors and spraying concrete at the bottom of the primary support to increase the strength of the surrounding rock or reduce the load.
3. The trend of the movement of the vault and surrounding rock to the tunnel clearance is evident, and the deformation is high. Therefore, monitoring and measurement should be strengthened to prevent invasion.

##### Three-bench method with a temporary invert

The numerical results of the three-bench method with a temporary invert indicates that this method increases the temporary invert, and its surrounding rock deformation control ability is improved relative to that of the bench cut method. However, the deformation is still high and the plastic zone is wider. The is due to the fact that the settlement of the vault caused by construction primarily occurs during the construction of the upper step. However, the temporary invert is placed at the bottom of the middle step. Hence, it cannot play an effective role. The recommended construction precautions are the same as those for the bench cut method.

##### Benching partial excavation method with a temporary invert

The numerical simulation results of the construction process of the benching partial excavation method with a temporary invert indicate that the control ability of the surrounding rock deformation is significantly improved relative to that of the bench cut method and three-bench method with a temporary invert. This is due to the fact that it places the temporary invert at the bottom of the upper step. Thus, the deformation of the surrounding rock and settlement of the vault can be controlled in time. Simultaneously, the core soil of the lower step provides effective support to the closed support system of the upper step.

The settlement of the vault due to the upper step construction accounts for approximately 89% of the final settlement. Therefore, settlement control of the upper step should be considered during this type of construction. Additionally, the soil in the middle of the lower step exhibits a good supporting effect on the supporting system, and protection should be strengthened during construction. Moreover, if necessary, shotcrete can be used for sealing.

##### Comparison of construction methods

Based on the perspective of the surrounding rock deformation, the double-sided pit method exhibits more section divisions and the best control effect on the surrounding rock deformation. The CRD method exhibits relatively large section divisions, and the settlement control effect is the second-best. The bench cut method exhibits the lowest ability to control deformation. The three-bench method with a temporary invert improves the control effect when compared with the bench cut method owing to the addition of the temporary invert. However, the disturbance of upper step excavation is an important part of its deformation causes, and it accounts for the largest proportion of the final deformation. Moreover, the placement of the temporary invert at the bottom of the middle step decreases its ability to provide the best deformation control. The benching partial excavation method with a temporary invert exhibits improvement over the three-bench method with a temporary invert, i.e., the temporary invert is placed at the bottom of the upper step to control the deformation of the surrounding rock in time. Consequently, its deformation control ability is significantly enhanced (Table 12).

**Table 12:**
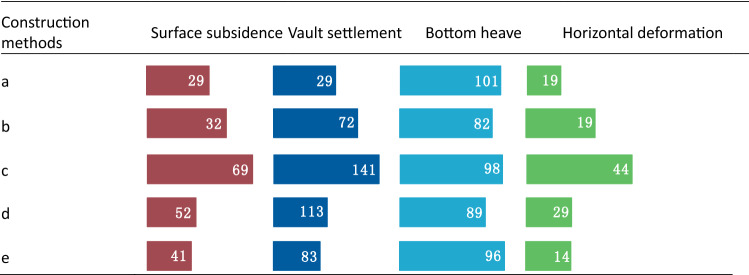
Final displacement of surrounding rock at different construction methods (unit: mm).

In the table, a denotes the double-sided pit method, b denotes the CRD method, c denotes the bench cut method, d denotes the three-bench method with a temporary invert, and e denotes the benching partial excavation method with a temporary invert.

In terms of the initial support force, the double-sided pit method and CRD method effectively control the surrounding rock deformation by reducing the excavation area and increasing the temporary support. Simultaneously, the internal force of the initial support increases and structural force becomes complex. In the bench cut method, the internal force of the structure is relatively low after the surrounding rock pressure is released to a certain extent.

In summary, the double-sided pit method and CRD method exhibit the best settlement control effects, highest construction safety, complex structural forces, and large internal forces. The settlement control effect of the benching partial excavation method with a temporary invert is next to the best, and the construction safety is average. The three-bench method with a temporary invert exhibits a poor settlement control effect and poor construction safety. Finally, the bench cut method exhibits the lowest settlement control effect and worst construction safety.

### Analysis of construction schemes

#### Different construction schemes

##### Advanced small pipe grouting (circular spacing 20 cm) + four-step method

At the beginning of the construction, a comprehensive analysis was conducted based on factors, including the tunnel geological conditions, construction period, and project investment. It was expected that the small pipe grouting combined with the four-step core soil method exhibits a low construction cost and fast excavation speed. Therefore, this scheme was preferred for construction purposes. However, through practical application, it was observed that this scheme is not suitable for aeolian sand formations. During construction, the sand leakage and sand sliding at the tunnel face and side walls were severe, thereby resulting in a funnel (approximately 2 m in diameter and 1.5 m in depth). There were several transverse cracks on the surface in front of the face. The crack spacing was in the approximate range of 0.5–1.0 m and widths were in the range of 2–4 cm. After excavation of the tunnel, the initial support settlement was excessively high, and the limit was severely invaded. The maximum intrusion limit was 90 cm, and the steel arch changed. The construction progress was very slow, and only 20 m was constructed for more than 3 months.

##### Advanced large pipe shed grouting + three-bench method with a temporary invert

In response to the problems in the construction, the advanced small pipe grouting was modified to advanced large pipe shed grouting. A comprehensive comparison of the double-sided pit method, CRD method, and bench method with a temporary invert revealed that although the double-sided pit method and CRD method can effectively control deformation, the construction cost is high and process is relatively complicated and slow. Therefore, based on the original bench cut method, a temporary invert was added, and the four steps were modified to a three-step excavation, i.e., the three-bench method with a temporary invert. This method exhibited the characteristics of a simple procedure and fast progress of the bench method and also added a temporary invert and enhanced the ability to control deformation.

##### Horizontal jet grouting piles + benching partial excavation method with a temporary invert

The large pipe shed was modified to horizontal jet grouting piles via a timely adjustment. The sand-fixing effects of the horizontal jet grouting piles were evident, and the phenomenon of sand leakage was effectively contained via practical application. Simultaneously, the three steps were adjusted to two steps: improving the initial support force of the upper step, reducing the initial support joints, increasing the vertical braces, and controlling the settlement and deformation of the vault in a timely manner. The actual application demonstrated the expected results.

#### Comparison of controlling deformation at different construction schemes

**Figure 15:**
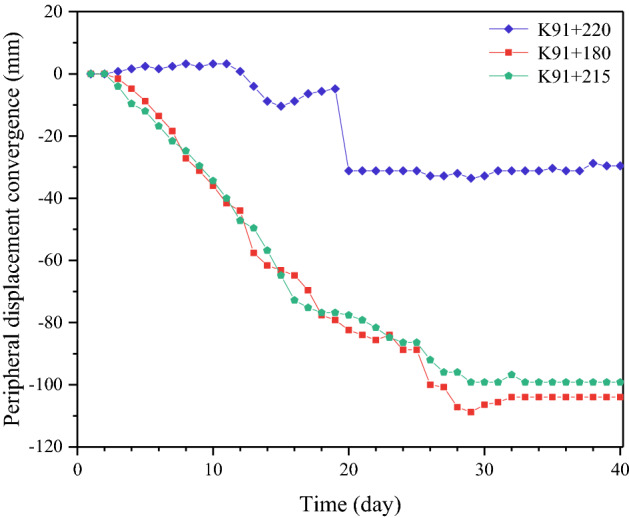
Curve of peripheral displacement convergence.

**Figure 16:**
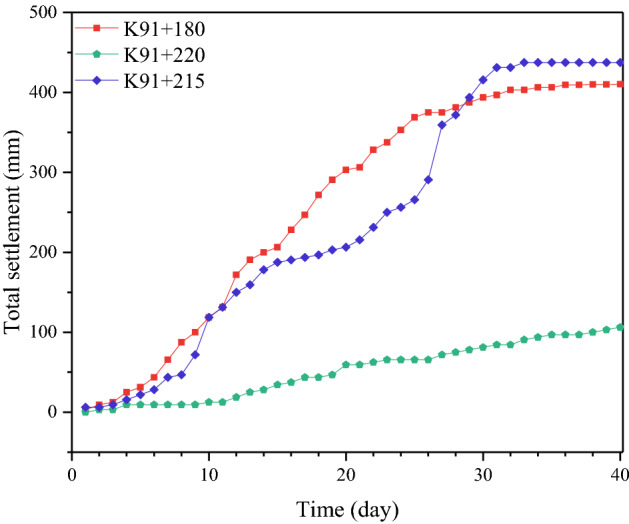
Curve of vault settlement.

Three representative tunnel sections were selected for the analysis of the deformation control ability (Table 13). The measured data with respect to the peripheral displacement convergence and vault settlement are shown in Figs. 15 and 16.

**Table 13:** General information of tunnel sections.

Mileage	K91 + 180	K91 + 215	ZK91 + 220
Construction schemes	Small pipe grouting + four-step method	Large pipe shed + three-bench method with temporary invert	Horizontal jet grouting pile + benching partial excavation method with temporary invert
Cover depth	29 m	29 m	29 m

The numerical comparison of the convergence and vault settlement indicates that the vault settlements and data for convergence due to the “small pipe grouting + four-step method” and “large pipe shed grouting + three-bench method with a temporary invert” are 3.9 times and 4.2 times and 3 times and 3.6 times those caused by the “horizontal jet grouting pile + benching partial excavation method with a temporary invert”, respectively.

Therefore, in the construction of aeolian sand tunnels, the “horizontal jet grouting pile + benching partial excavation method with a temporary invert” can be considered as the optimal scheme with respect to the control of settlement and deformation. Furthermore, this scheme exhibits evident advantages relative to the other two schemes.

### Suggestions for tunnel construction in aeolian sand strata

Based on the research in this study, the following suggestions are provided.

#### Advanced support

Horizontal jet grouting piles should be adopted for advance support. When conditions are restricted, such as by capital or the construction period, under the premise of ensuring the grouting effect, advanced large pipe shed grouting can be selected. The advanced small pipe grouting method should be used as a supplement to solve the problems of sand leakage due to the increase in the spaces between pipe sheds owing to the progress of the pipe shed system. Furthermore, when advanced small pipe grouting is used as the primary advanced support method, it should be arranged in a dense configuration or in the double-layer form.

#### Construction method

In the case of strict control requirements for surface subsidence, the double-sided pit method or CRD method should be adopted. Furthermore, when there is no strict control requirement for the surface subsidence, it should be recommended to adopt the benching partial excavation method with a temporary invert. This method exhibits few excavation steps and flexible construction systems, which can control the settlement and deformation of the vault in time. Hence, the three-bench temporary invert method and bench method without a temporary invert should not be used.

## Conclusions

In this study, we considered Shenmu No. 1 tunnel as a case study, investigated the engineering characteristics of aeolian sand tunnels, compared the grouting effects of commonly used grouting materials, and discussed the reinforcement effects of different construction schemes in aeolian sand tunnels. The specific conclusions are listed as follows.

1. Ordinary cement grout exhibits a large particle size, and it is difficult to inject aeolian sand layers with a pore size of 10 μm. Moreover, the cement particles are suspended in the slurry. Even if the particle sizes are smaller than the pores of the sand layer, the filtration of the sand layer leads to an extremely small permeability range, and the grout can consolidate on the surroundings of the grouting holes.
2. The superfine cement grout exhibits a small particle size with an average particle size of 4 μm. Theoretically, it can be injected into an aeolian sand layer with a pore size of 10 μm, but actual field tests show that the grouting effect does not significantly differ from that of ordinary cement. This is due to the fact that sedimentation is prone to occur, which in turn results in poor stability.
3. The modified sodium silicate grout exhibits good permeability. However, the gel time is short, blockage in the pipe often occurs in the test, and the proportioning process is more complex. After grouting, the strength of the reinforced body is low (0.2 MPa), and it can easily weather and break after exposure.
4. Horizontal jet grouting piles should be adopted for advance support. To ensure the grouting effect, advanced large pipe shed grouting should be selected, and the advanced small pipe grouting method should be used as a supplement.
5. In the case of strict control requirements for surface subsidence, the double-sided pit method or CRD method should be adopted. Furthermore, when there is no strict control requirement for surface subsidence, the benching partial excavation method with a temporary invert should be adopted. There are few excavation steps and flexible construction systems that can control the settlement and deformation of the vault in time. Finally, the three-bench temporary invert method and bench method without a temporary invert should not be used.
